# Experimental hut evaluation of linalool spatial repellent agar gel against *Anopheles gambiae sensu stricto* mosquitoes in a semi-field system in Bagamoyo, Tanzania

**DOI:** 10.1186/s13071-014-0550-2

**Published:** 2014-12-05

**Authors:** Mgeni Mohamed Tambwe, Edgar Mtaki Mbeyela, Brian Migamyo Massinda, Sarah Jane Moore, Marta Ferreira Maia

**Affiliations:** Ifakara Health Institute, Intervention and Environmental Health and Ecological Sciences, P.O. Box 74, Bagamoyo, Tanzania; Swiss Tropical & Public Health Institute, Soccinstraße 57, 4002 Basel, Switzerland; University of Basel, Petersplatz 1, 4003 Basel, Switzerland

**Keywords:** 73% d-linalool agar, Semi-field system, *Anopheles gambiae* s.s., Experimental hut

## Abstract

**Background:**

Malaria vector control is in need of new tools to face its current challenges such as the spread of pyrethroid-resistance and the increase of outdoor feeding mosquitoes. New strategies such as spatial repellents need to be evaluated as supplemental tools to existing control measures such as insecticide treated bed nets and indoor residual spraying. Linalool is a naturally occurring terpene alcohol commonly found in flowers and spices with reportedly repellent properties.

**Methods:**

Four experimental huts fitted with exit traps and enclosed inside a large screened semi-field system were used for the evaluation. The tested spatial repellent product consisted of an agar gel emanator containing 73% linalool. Two rounds of experiments using a Latin square design were conducted to evaluate the efficacy of the linalool emanators compared to no treatment (negative control) and a transfluthrin coil (positive) against lab-reared disease free *Anopheles gambiae* s.s.. The emanators were hung inside experimental huts where two volunteers were sleeping unprotected. The outcome measures were repellency, % feeding inhibition, %mortality and post 24 h % mortality.

**Results:**

Unlike the mosquito coil, the linalool emanators did not show any feeding inhibition, repellency or induced mortality compared to the negative control. On the other hand mosquitoes kept for 24 h post exposure were 3 times more likely to die after being exposed to two 73% linalool emanators than the negative control.

**Conclusions:**

Our results indicate that linalool agar gel emanators are not adequate as a spatial repellent against *Anopheles gambiae* s.s.. However adding linalool to known repellent formulations could be advantageous, not only because of its pleasant scent but also because of the delayed mortality effect it has on mosquitoes.

## Background

In tropical and sub-tropical regions vector-borne diseases spread by mosquitoes such as malaria remains a serious public health concern. National malaria vector control programs have mainly been focused on insecticide-treated nets (ITNs) and indoor residual spraying (IRS), which led to a significant reduction across endemic countries [1]. However these intra-domiciliary measures are insufficient to reach elimination and there is call for new and innovative tools for malaria vector control [2].

Bed-nets have a recognized impact on malaria control and in areas where malaria mosquitoes bite earlier in the evening, repellents can also help reduce malaria transmission [3,4]. Various chemicals have been deployed to control malaria vectors, these include synthetic insecticides/repellents and also plant based repellents [3,5]. Synthetic pyrethroid are currently the only chemicals recommended by World Health Organization Pesticide Scheme (WHOPES) for net impregnation and indoor residual spraying because they show low mammalian toxicity and fast acting properties against mosquitoes [6]. However, pyrethroid-resistance is present and spreading in many areas of Africa [7], by limiting the chemicals toxic effect it reduces the effectiveness of ITNs or IRS and severely compromises advances achieved in the fight against malaria [8]. The spread of resistance unfortunately outpaces the development of new, safe insecticides and so the reliance of vector control programs on pyrethroids becomes a ticking bomb.

Personal protection against mosquito bites can be achieved by using bed-nets when people are sleeping or by using repellents while people are active. The use of repellents is wide spread among travelers to tropical regions and has been found to reduce malaria incidence in areas where mosquitoes bite earlier in the evening [3,4]. Repellents can be used topically or spatially. Topical repellents are applied directly onto the skin, require regular compliance by the user and offer only individual protection. In contrast spatial repellents create a protective area by volatilizing repellent into the air and so providing protection for multiple individuals within a given radius. The most commonly used spatial repellent is the mosquito coil, which acts by dispersing pyrethroid volatiles into the air through slow combustion. Similarly to topical repellents, this intervention requires nightly compliance as well as regular purchasing. Another type of spatial repellents are known as emanators. Emanators incorporate repellent chemicals with low vapor phase into a substrate such as paper or agar-gel, which enables a passive dispersion of repellent volatiles at ambient temperature. Unlike topical repellents and mosquito coils, emanators do not require compliance and may be effective over an extended period of time depending on the formulation.

Linalool is pleasant-scented naturally occurring terpene alcohol commonly found in flowers and spices. It has been isolated from a range of plants that are traditionally used to repel mosquitoes by local African communities such as *Ocimum forskolei*, *Mkilua fragrans and Thymus vulgaris* [9-11]. Studies investigating the electrophysiological effect of repellents on mosquito olfactory receptors discovered that the same odor receptors that respond to DEET also respond to linalool in *Culex quinquefasciatus* [12]. Human forearm bioassays performed using *Culex pipiens pallens* showed that linalool can provide up to 92% protection from bites for around 1 hour [11]. In addition olfactometric studies measuring spatial repellency responses of *Stegomyia aegypti* revealed that a combination of linalool and dehydrolinalool provided 33.6% more spatial repellency compared to control [13]. The spatial repellency properties of linalool have been tested in the field in the format of an oil candle [14]. Repellency against wild mosquitoes in an Israeli oasis was measured at nearly 65% when the linalool candle was placed at 1 m distance from the human. Given the literature indicating the potential of linalool as a spatial repellent, we proposed to investigate the behavioral responses of *Anopheles gambiae* s.s. when exposed to linalool agar gel emanators in semi-field conditions.

## Methods

### Semi-field system

**Figure 1:**
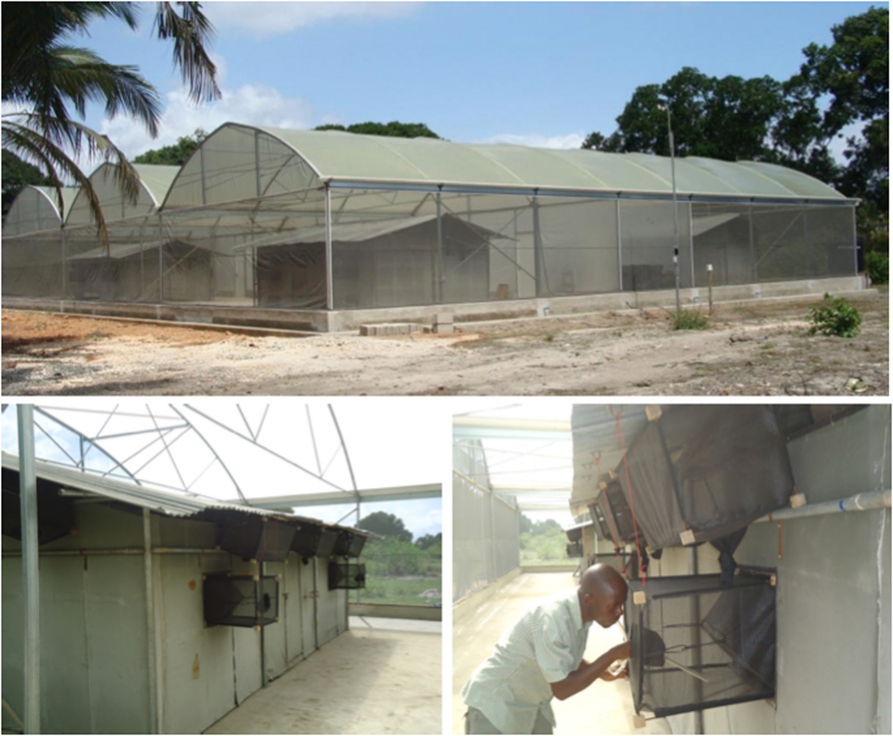
**Semi-field system and experimental hut at the Ifakara health Institute, Bagamoyo, Tanzanaia.**

The experiment was conducted in a fully screened Semi-field System (SFS) 22 × 29 m situated in Bagamoyo, Tanzania (Figure 1). The climatic conditions (temperature, light, humidity, wind speed) within the SFS were equal to outdoor conditions. The whole semi-field system rested on concrete surrounded by a narrow water-filled channel to exclude ants and other predators. SFSs provide a disease-free, controlled environment whereby experiments including human landing catches can be performed faster and more efficiently than in the field [15]. The SFS is divided into two compartments separated by a corridor. In each compartment two moveable experimental huts were setup.

### Experimental huts

The experimental huts were designed resembling a typical Tanzanian household in terms of size, structure and mosquito exit/entry points (eaves, windows and doors). Mosquito exit traps were fitted to all the openings of the experimental huts. Two mattresses were placed in the middle of the huts.

### Mosquitoes

Disease-free and blood-naïve female *Anopheles gambiae* s.s. (Ifakara strain), aged 2-5 days old were used in this study. These mosquitoes were reared under natural photoperiod at 27°c and 80% humidity, in Kingani insectary laboratory of the Ifakara Health Institute. The mosquitoes were starved of water and sugar for a period of 6 hours before experiments. The mosquitoes for the experiment were released at the middle of the experimental hut by the volunteers.

### Linalool agar-gel emanators

**Figure 2:**
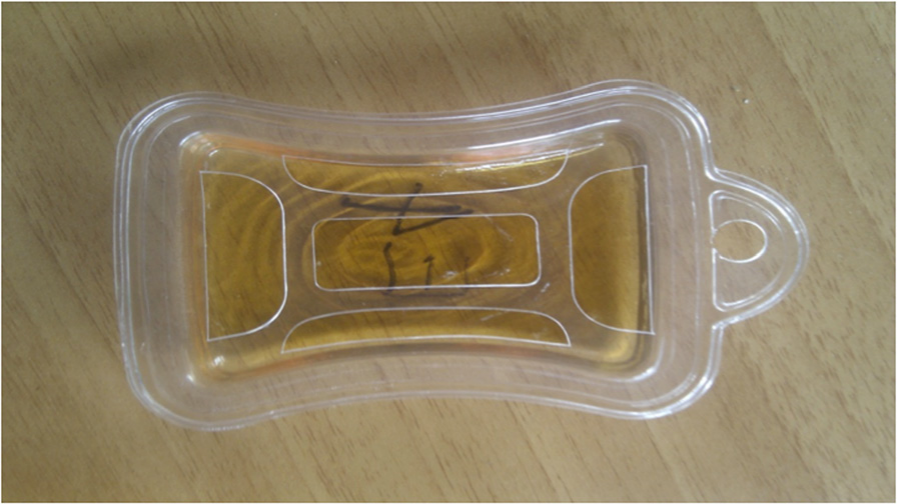
**Linalool 73% agar gel emanator.**

The spatial repellent tested in this study consisted of an agar gel air freshener containing 73% d-linalool isomer held in a plastic case (Figure 2) with approximately 40 cm^2^ of surface.

### Volunteers

The volunteers sleeping inside the huts were recruited upon signed written informed consent. They were provided with a mattress and instructed not to consume alcohol or smoke at least 5 hours prior to the experiments as well as to avoid deodorants and colognes during the study period. The volunteers were asked to wear protective clothing exposing only their legs to prevent generalized discomfort caused by mosquito bites. The weather was warm thus volunteers slept without blankets and were not provided with a bed-net since one of the objectives of study was to investigate the feeding behavior of *Anopheles gambaie* s.s when exposed to d-linalool emanators. All volunteers were African men between 25 and 40 years of age. The same volunteers were used throughout the entire experiment.

### Experimental design

Three treatments were compared: 1) 73% d-linalool emanators, 2) 0.03% transfluthrin mosquito coils (positive control) and 3) no treatment (negative control). Two rounds of experiments using a Latin square design were performed:

1. Experiment 1 – 4 × 4 Latin square designs (8 nights). A total of four experimental huts were used to test three treatments: 1) two 73% d-linalool emanators, 2) two 0.03 % transfluthrin coils (positive control) and 3) no treatment (negative control). Two huts from separate SFS compartments were assigned the same treatment consisting of two 73% d-linalool emanators hanging approximately 1.5 m high above the ground and 1 m from each corner, these emanators were fixed throughout experimental night. The other two huts were randomly allocated two transfluthrin 0.03% mosquito coils lit on plates placed in two mid points of the hut and one hut was left with no treatment. In both experimental huts, two participants slept on a mattress at the center of the hut with.
2. Experiment 2 – 3 × 3 Latin square designs (9 nights). A total of three experimental huts were used to test three treatments: 1) four 73% d-linalool emanators, 2) 0.03% transfluthrin coils (positive control) and 3) no treatment (negative control). Treatments were randomly allocated to each hut. Four emanators were hung in each quadrant of the hut at approximately 1.5 m high above the floor. Two 0.03% transfluthrin mosquito coils were lit by the technicians and placed on plates in two mid points of the hut. One hut was left with no treatment.

During each experimental night two volunteers slept unprotected inside each experimental hut. Each day the volunteers were rotated between huts and the treatments remained fixed using a Latin Square design. At 19:00 fifty lab-reared starved *Anopheles gambiae *s.s. were released inside each experimental hut and allowed to feed freely on the volunteers sleeping inside the huts. Mosquitoes were collected from inside exit traps at 00:00, 04:00 and 8:00 using mouth aspirators. At 8:00 the huts walls and floors were aspirated using Prokopack aspirators to collect resting mosquitoes [16].

The collected mosquitoes were placed in paper cups labeled with the hut number, time of collection and collection site (exit trap, wall or floor). The cups contained cotton wool soaked with sucrose solution to prevent mortality unrelated to the exposure to the treatments. In the morning mosquitoes were counted and recorded as dead or alive as well as fed or unfed. Dead mosquitoes were discarded; the remaining collected were placed in an appropriately labeled cup and taken to the insectary for observation of mortality 24 hours post-exposure. These mosquitoes were kept on 10% glucose soaked cotton wools in normal insectary conditions. After 24 hours, mosquitoes were observed for mortality.

### Outcome measures and statistical analysis

The following outcomes were measured: feeding inhibition, repellency, mortality and 24 h mortality post exposure.

- Feeding inhibition was measured by comparing the proportion of collected fed mosquitoes in the treatments to the negative control.
- Repellency was measured by comparing the proportion of mosquitoes collected in exit traps in the treatments to negative control.
- Mortality was measured by comparing the proportion of dead mosquitoes found in each treatment to the negative control.
- Mortality 24 h post exposure was measured by comparing the proportion of dead mosquitoes post 24 h exposed to the treatments compared to the negative control.

In order to analyze the proportional data 0.01 was added to all the data values to avoid errors caused by divisions by 0. The data were analyzed in STATA 11, using generalized linear models (GLM) with logit function, fitted to a binomial distribution with robust errors. The dependent variables used were “proportion fed” for feeding inhibition, “proportion dead” for mortality and “proportion dead post 24 h exposure”, the fixed effect was treatment and random effects were collectors, day, and volunteers. P-value and odds ratio were used to describe the difference between data compared to the negative control hut.

Repellency was analyzed using Kaplan-Meier survival analysis; the occurrence of event was classified as a mosquito exiting the hut. The exit behavior of *Anopheles gambiae* s.s. was compared between each treatment.

### Ethical clearance

The volunteers were given an information sheet describing the objectives, study procedures, risks and benefits of their participation in this study. A written informed consent was obtained from individual volunteer before the experiments. The study was approved by the Ifakara Health Institute Ethical Review Board (ref. number IHI- IRB No.A 019 2007).

## Results

### Experiment 1

Table 1 below summarize the results of repellency activity of linalool agar gel. During the 8 experimental nights a total of 1021 female *An. gambiae* s.s. were recovered out of 1600 released mosquitoes. The 0.03% transfluthrin + coils inhibited feeding by 82% (OR = 0.27; 95% CI = [0.11 – 0.64]; p = 0.003) while the control only 10% of the mosquitoes didn’t feed. Two 73% d-linalool agar gel emanators did not inhibit feeding as 66% (OR = 1.37; 95% CI = [0.65 – 2.78]; p = 0.427) of the mosquitoes collected had fed on the volunteers. This difference in the feeding status of mosquitoes in the test as compared to the control is by chance and not statically significant. Mosquitoes were over six times more likely to have been found dead in the huts where the transfluthrin coils were burning compared to negative control (OR = 6.37; 95% CI = [2.40 – 16.87]; p < 0.001). Mosquito mortality in the huts with two 73% d-linalool agar gel emanators was only slightly higher than in the control and was not statistically significant (OR = 1.78; 95% CI = [0.70 – 4.54]; p = 0.23). On the other hand mosquitoes exposed to linalool were 3 times more likely to die after 24 hours compared to control (OR = 2.93; 95% CI = [1.16 – 739]: p-value = 0.022).

**Table 1:** **Mosquitoes behavior following exposure to linalool agar gels using a 4x4 Latin square design**

**Hut treatments**	**Total released**	**Total recovered**	**Total fed**	**Odds ratio**	**CI 95%**	**P-** **value**	**% Feeding success**
Negative control	400	248	223	1	-	-	90%
Two 0.03% Transfluthrin coil	400	237	43	0.27	(0.11-0.64)	0.003	18%
Two linalool emanators	800	536	350	1.34	(0.65-2.78)	0.427	66%
			**Total dead**				**% Mortality**
Negative control	400	248	12	1	-	-	5%
Two 0.03% Transfluthrin coil	400	237	170	6.37	(2.40 – 16.87)	<0.001	72%
Two linalool emanators	800	536	64	1.78	(0.70 – 4.54)	0.23	12%
	**Total alive**	**Total dead post 24 h**					**% Mortality 24 hrs**
Negative control	237	25		1	-	-	11%
Two 0.03% Transfluthrin coil	65	12	-	1.65	(0.4 4–6.21)	0.46	18%
Two linalool emanators	275	57	-	2.93	(1.16-7.39)	0.022	21%

Odds ratio, 95% confidence intervals and p-values were obtained from statistical analysis using generalized linear models. Data from the linalool treated huts were aggregated; all treatments were compared to the negative control.

### Experiment 2

**Figure 3:**
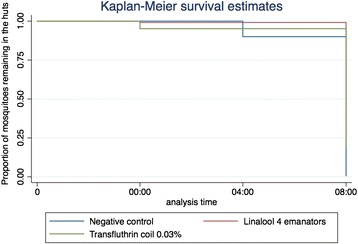
**Kaplan**-**Meier survival graph representing the time at which mosquitoes exit the different treatment huts.**

Table 2 shows the summary of the results obtained from repellent activity of linalool in 3 × 3 experimental design. During the 9 experimental nights a total of 957 female *An. gambiae s.s*. were recovered out of 1350 released mosquitoes. Similarly to Experiment 1, in this round of experiments four 73% d-linalool agar gel emanators did not inhibit mosquitoes from feeding on the volunteers inside the experimental huts compared to no treatment (OR = 0.7; 95% CI = [0.34 – 1.33]; p = 252). Only 4% of mosquitoes inside the hut where 0.03% transfluthrin coils were lit succeeded in obtaining a blood meal from the volunteers (OR = 0.001; 95% CI = [0.0004 – 0.002]; p < 0.001). Also in this hut, mosquitoes were 221 times more likely to die due to exposure to the transfluthrin volatiles compared to control (OR = 221; 95% CI = [88.84 – 553.2]; p = 0.023). There was no difference between the number of collected dead mosquitoes in the hut treated with four 73% d-linalool agar gel emanators and the control (OR = 0.9; 95% CI = [0.195 – 4.340]; p = 0.252). Mosquitoes exposed to d-linalool and transfluthrin presented slightly higher 24 delayed mortality, 24 and 9% respectively compared to control (7%) but were not statistically significant. In terms of repellency, mosquitoes in the control hut did not exit until after midnight while mosquitoes exposed to transfluthrin were more likely to leave earlier in the evening (Figure 3). Linalool did not induce a repellence effect on the mosquitoes.

**Table 2:** **Mosquitoes behavior following exposure to linalool agar gels using a 3x3 Latin square design**

**Hut treatments**	**Total released**	**Total recovered**	**Total fed**	**Odds ratio**	**CI 95** **%**	**P-** **value**	**% Feeding success**
Negative control	450	372	358	1	-	-	96%
Two 0.03% Transfluthrine coil	450	238	10	0.001	(0.0004-0.002)	<0.001	4%
Four linalool emanators	450	347	331	0.7	(0.34-1.33)	0.252	95%
			**Total dead**				**% Mortality**
Negative control	450	372	9	1	-	-	2%
Two 0.03% Transfluthrine coil	450	238	238	221	(88.84 – 553.2)	0.023	100%
Four linalool emanators	450	347	7	0.9	(0.195 – 4.340)	0.252	2%
	**Total alive**	**Total dead post 24 h**					**% Mortality 24 hrs**
Negative control	363	26	-	1	-	-	7%
Two 0.03% Transfluthrin coil	45	11	-	1.79	(0.34 – 9.55)	0.49	24%
Four linalool emanators	340	31	-	1.76	(0.36-0.483)	0.483	9%

Odds ratio, 95% confidence intervals and p-values were obtained from statistical analysis using generalized linear models. Data from the linalool treated huts were aggregated; all treatments were compared to the negative control.

## Discussion

Plants extracts containing monoterpene oils such as linalool have been shown to possess a natural repellence effect against various mosquito species [11,14,17]. For centuries traditional practices have been using plants by burning and smouldering of leaves or flowers in order to achieve protection from mosquito bites [18]. Findings from previous studies have shown that linalool in different formulations repel mosquitoes. In this study we have reported that 73% d-linalool agar emanators do not inhibit feeding of *Anopheles gambiae* s.s. and do not kill mosquitoes exposed to its volatiles. Also, 73%-linalool agar gel emanators do not drive mosquitoes to leave the experimental huts showing no repellent effect. Moreover, we did not see any dose-dependent effect after doubling the number of emanators in each hut. On the other hand, mosquitoes caught in huts treated with linalool were 3 times more likely to die 24 h post collection than in the control. This may be because linalool is a reversible inhibitor of acetyl cholinesterase, capable of disrupting neurotransmission in insects and inducing knock-down [19]. These different findings could be explained by the differences in methodology, formulation, and percentage of linalool used in each study.

## Conclusion

We concluded that the tested 73% d-linalool agar gel emanators do not provide protection against malaria vectors. More studies should be performed using other formulations and perhaps repellent blends containing linalool. If effective, emanators could provide a pleasant household protection method, with high compliance.
